# The relationship between parental emotional warmth and school bullying: The chain mediation role of social support and emotional regulation

**DOI:** 10.1371/journal.pone.0323261

**Published:** 2025-05-07

**Authors:** Zhengwei Wan, Shuyu Huang

**Affiliations:** College of Teachers, Chengdu University, Chengdu, Sichuan, China; Universidad Santiago de Cali, COLOMBIA

## Abstract

This study explores the complex interplay between parental emotional warmth, social support, emotional regulation, and school bullying among students. Using a chain mediation model, we investigate how parental emotional warmth indirectly influences school bullying through the mediating roles of social support and emotional regulation. A sample of 566 students aged 10–13 participated in this study. The results indicate that parental emotional warmth significantly enhances social support, which in turn positively influences emotional regulation, thereby reducing school bullying behaviors. However, the direct effect of parental emotional warmth on school bullying was not significant. These findings highlight the importance of social support and emotional regulation as mechanisms through which parental emotional warmth affects school bullying. The study contributes to the theoretical understanding of the impact of family dynamics on children’s behavior and provides practical insights for developing multi-faceted interventions to prevent and reduce school bullying. Future research should employ longitudinal designs and diverse populations to further validate these findings and explore cultural variations.

## Introduction

School bullying is a prevalent and increasingly serious issue in today’s educational field, significantly impacting students’ mental health and academic performance. Research shows that students who experience bullying are more likely to develop psychological problems such as anxiety, low self-esteem, and depression, with severe cases possibly leading to suicide [1,2]. Therefore, exploring the factors influencing bullying behavior, especially family factors, is of great theoretical and practical significance.

Parental upbringing, as a key factor in the family environment, has a profound impact on students’ behavior and psychological state. Studies indicate that parental upbringing not only directly affects students’ behavior but also indirectly influences their performance at school through emotional regulation and social support [3,4]. This paper aims to investigate the relationship between parental upbringing and school bullying, focusing on the chain mediation role of social support and emotional regulation.

### Theoretical framework

This study’s theoretical framework is based on Bronfenbrenner’s social ecological systems theory [5], which places individuals in a multi-level environment, exploring the comprehensive effects of environmental factors at different levels on individual behavior. This theory posits that individual development and behavior are influenced by the microsystem, mesosystem, exosystem, and macrosystem. At the microsystem level, parental upbringing directly affects children’s behavior and emotional regulation abilities. Authoritative parenting, through providing emotional support and behavior norms, helps children develop positive emotional regulation strategies and social interaction patterns, thus reducing bullying behavior [6]. At the mesosystem level, cooperation between family and school can provide a more comprehensive support system for children, promoting their psychological health and social adaptability [7]. The exosystem includes family and school policies and environments that indirectly influence children’s behavior and emotional regulation abilities. The macrosystem encompasses cultural and societal values that also significantly impact parenting styles and school bullying behaviors [5].

### The relationship between parental emotional warmth and school bullying

Parental emotional warmth plays a crucial role in children’s growth. Studies indicate that parental emotional warmth can effectively reduce the occurrence of school bullying behaviors. A warm parenting environment makes children feel loved and cared for, which enhances their self-esteem and self-worth, reducing the likelihood of engaging in bullying behaviors [8,9]. Additionally, parental emotional warmth improves children’s emotional regulation abilities, enabling them to better handle conflicts and stress, thus avoiding bullying others to release negative emotions [10].

Warm parenting also promotes positive interactions with peers, fostering empathy and cooperation. These social skills help children interact better with others in school, reducing the chances of bullying [3]. Research also shows that parental emotional warmth can reduce children’s negative emotional responses when bullied, enhancing their coping abilities and resilience [11].

Further studies emphasize the long-term relationship between parenting styles and children’s behavior. For instance, studies have shown that parental emotional warmth and monitoring levels significantly affect children’s aggressive behavior. Warm and structured parenting reduces the occurrence of aggressive behavior, especially during adolescence [2,12]. Similarly, positive parental involvement and effective communication reduce children’s problematic behaviors in school, including bullying.

Conversely, emotional warmth in parenting can reduce children’s hostility and increase their empathy, lowering their likelihood of participating in bullying. Research shows that children with high empathy are better at understanding others’ feelings and needs, thus less likely to engage in harmful behaviors [13]. Conversely, children lacking emotional warmth may exhibit higher hostility and aggression, increasing their risk of bullying [14].

### The mediating role of social support

Social support plays a crucial mediating role between parental emotional warmth and school bullying. Social support can be divided into family support, peer support, and school support. Studies show that adequate social support helps children build a positive self-concept and good mental health, reducing the likelihood of bullying behaviors [15,16].

In terms of family support, parental emotional warmth enhances children’s sense of belonging and security, enabling them to seek family help and support when facing bullying [17]. Peer support provides emotional comfort and practical help, allowing children to receive understanding and support from peers when bullied, reducing the negative psychological impact of bullying [18]. School support includes teacher care and anti-bullying policies, creating a safe and supportive school environment, reducing bullying incidents [19].

Further research shows that different types of social support have varying mechanisms of action in children and adolescents. For example, Wentzel found that teacher support and peer support have different effects on student behavior at different developmental stages [20]. Teacher support significantly impacts behavior norms and emotional support in early education, while peer support is more important for emotional support and social interaction in adolescence.

Moreover, social support can directly reduce bullying behaviors and indirectly reduce bullying by enhancing children’s psychological resilience. Research shows that children with high psychological resilience can better adjust their emotions and behaviors when facing bullying, reducing long-term psychological harm [21]. This resilience can be fostered and enhanced through comprehensive support from family, school, and peers.

### The mediating role of emotional regulation

Emotional regulation refers to individuals’ ability to regulate and control their emotional responses when facing stress or negative emotions. Studies indicate that emotional regulation also mediates the relationship between parental emotional warmth and school bullying [22,23]. Parental emotional warmth helps children develop good emotional regulation abilities, enabling them to adopt positive coping strategies when facing conflicts and stress instead of bullying others to release negative emotions [24].

Good emotional regulation abilities allow children to handle interpersonal relationships better in school, reducing conflicts with peers and thus reducing bullying incidents [25]. Research also shows that enhancing children’s emotional regulation abilities increases their psychological resilience, helping them maintain emotional stability when bullied, reducing psychological health problems [23].

The development of emotional regulation abilities relies not only on parenting styles but also on personal traits and environmental factors. For instance, children with high emotional intelligence can effectively use emotional regulation strategies when facing stress, reducing the accumulation and outburst of negative emotions [26]. Moreover, the school environment and peer interactions significantly impact children’s emotional regulation abilities. A positive school atmosphere and good peer relationships provide emotional support, helping children better manage their emotions [27].

### The chain mediation role of social support and emotional regulation

Social support and emotional regulation play a chain mediating role in the relationship between parental emotional warmth and school bullying. Specifically, parental emotional warmth enhances children’s social support, providing them with more emotional support and practical help, thereby improving their emotional regulation abilities [28]. This chain mediation process can be explained as follows: Parental emotional warmth → Social support → Emotional regulation → School bullying [29,30].

Firstly, parental emotional warmth enhances children’s social support, allowing them to obtain more support resources when facing school bullying. These support resources provide emotional comfort and help children develop good emotional regulation strategies [28]. Secondly, good emotional regulation abilities enable children to adopt positive coping strategies when facing conflicts and stress, reducing the likelihood of releasing negative emotions through bullying others [31]. Ultimately, this chain mediation process results in children exhibiting fewer bullying behaviors in school, promoting a harmonious and safe school environment [32,33].

This research on chain mediation further reveals the interaction mechanisms between family, school, and peers in children’s development. For example, Bowes et al. [30] found that children with high family and school support exhibit higher emotional regulation abilities and psychological resilience when facing bullying, reducing bullying behaviors. Similarly, Rueger et al. [16] found that peer support plays a crucial role in alleviating the emotional and behavioral impact of bullying on children.

Additionally, the interaction mechanism of social support and emotional regulation reflects the multi-level nature of psychological health interventions. Enhancing the support network from family, school, and peers can effectively improve children’s emotional regulation abilities, reducing the occurrence and negative impact of bullying behaviors [5]. This suggests that interventions against school bullying should be comprehensive, focusing on improving parenting styles and enhancing support from both schools and peers, ultimately creating a multi-level support system to safeguard children’s psychological health.

### Current study

The current study aims to explore the complex interplay between parental emotional warmth, social support, emotional regulation, and school bullying among students. By constructing a chain mediation model, this study investigates how parental emotional warmth indirectly influences school bullying through the mediating roles of social support and emotional regulation. This research extends previous studies by not only examining the direct effects of parental emotional warmth on bullying behavior but also by elucidating the pathways through which this effect occurs. Understanding these mechanisms can provide valuable insights for developing effective interventions to reduce school bullying. The primary research question guiding this study is: How does parental emotional warmth affect school bullying, and what are the mediating roles of social support and emotional regulation in this relationship?

## Methodology

The research was conducted in accordance with the principles stated in the Declaration of Helsinki for experiments involving humans. After the researcher received approval from the presidents of three primary schools in southwest China (2024, Apr 5) and the Research Ethics Review Committee in China (2024-23; 2024, Apr 7), a written parental consent form was sent to the president via email to share with potential participants. This consent form provided a description of the study’s goals, data collection procedures, potential participation risks, and the researcher’s contact information (i.e., telephone and email). All data were collected using paper-based questionnaires, which were administered under supervised conditions to ensure the reliability of responses. The research only used data from students who had signed the parental consent form. The study was completed between April 10, 2024, and April 30, 2024.

### Participants

Using convenience sampling, questionnaires were administered to 566 students, yielding valid responses with a response rate of 97.2%. Among them, 275 were male (48.6%) and 291 were female (51.4%). The age range was 10–13 years, with an average age of 11.092 ± 0.552 years. The survey was conducted with the informed consent of the students, and personal information was kept strictly confidential.

To further explore the internal mechanisms by which parental emotional warmth affects students’ school bullying, this study treats parental emotional warmth as the independent variable, social support and emotional regulation as mediating variables, and school bullying as the dependent variable, constructing a chain mediation model of social support and emotional regulation between them. The study uses Model 6 of the PROCESS macro program to analyze the chain mediation role of social support and emotional regulation, applying Bootstrap repeated sampling 5000 times and calculating the 95% confidence interval of the mediation effect.

### Instruments

The present study adapted the Chinese version of the s-EMBU-C questionnaire, developed by Jiang Jiang et al. [34]. The Parental Emotional Warmth Scale used in this study selected seven items from the emotional warmth dimension of the s-EMBU-C questionnaire. This scale uses a 4-point Likert scoring system, ranging from 1 indicating “never” to 4 indicating “always.” The rationale for selecting these seven items was based on their strong psychometric properties, particularly their construct validity and relevance to the target population (minors). The selection of this dimension was also supported by the good structural validity of the s-EMBU-C questionnaire, of which the Parental Emotional Warmth Scale is one of three subscales. In this study, the Cronbach’s alpha coefficient for the Parental Emotional Warmth Scale was 0.814.

The Emotional Regulation Questionnaire (ERQ) developed by Gross [35], with Cognitive Reappraisal and Expression Suppression dimensions, consists of 10 items scored on a 7-point scale, with higher scores indicating a higher frequency of using emotional regulation strategies. The Cronbach’s alpha coefficients for the Cognitive Reappraisal and Expression Suppression dimensions in this study are 0.796 and 0.725, respectively.

The Social Support Scale (SSS) developed by Blumenthal et al. [36] and later translated and modified by Jiang Qiankun, forms a Chinese version. PSSS contains 12 self-assessment items, each using a 1–7 Likert scale, ranging from strongly disagree to strongly agree. Higher total scores indicate higher individual social support. The Cronbach’s alpha coefficient of this scale in this study is 0.918.

The Chinese version of the Olweus Bully Questionnaire, revised by Zhang [37], consists of 6 items scored on a 5-point scale, ranging from “never happened this semester” to “several times a week.” Higher scores indicate higher levels of bullying victimization. The Cronbach’s alpha coefficient of this scale in this study is 0.793.

### Data processing and statistical analysis

Using SPSS 26.0 software and the PROCESS macro (Hayes, 2013) for statistical analysis of collected data. The Harman single-factor method was used to test for common method bias, and Pearson correlation analysis was used to explore the relationships between major variables. Additionally, Model 6 of the PROCESS macro was used to analyze the chain mediation role of social support and emotional regulation.

To explore the internal mechanism of how parental emotional warmth affects school bullying, this study takes parental emotional warmth as the independent variable, emotional regulation and social support as mediating variables, and school bullying as the dependent variable, constructing a chain mediation model of emotional regulation and social support between parental emotional warmth and school bullying. The study uses Model 6 of the PROCESS macro program to analyze the chain mediation role of emotional regulation and social support, using Bootstrap repeated sampling 5000 times and calculating the 95% confidence interval of the mediation effect. If the interval does not include 0, the mediation effect is significant; if it includes 0, the mediation effect is not significant [38].

In terms of data assumptions for the PROCESS macro analysis, the normality of variables was tested using skewness and kurtosis, and the results showed some deviation from normality, which is a common occurrence in psychological research with complex constructs. Additionally, the assumption of independence was met as the data were collected from distinct individual participants without any clustering or repeated measures. To address this, non-parametric methods such as Bootstrap sampling (5000 iterations) were employed to reduce the impact of non-normality.

For handling missing data, multiple imputation was applied to fill in any missing values. This method was chosen to avoid potential biases introduced by listwise deletion and to ensure a more accurate representation of the data set. After imputation, the analyses were conducted on the complete dataset to ensure robustness of the findings.

## Results

### Common method bias test

Using Harman’s single-factor test method to test for common method bias. The results showed that there were five factors with eigenvalues greater than 1, and the variance explanation rate of the first common factor was 32.594%, which is less than the critical value of 40%, indicating that there is no obvious common method bias in this study [39], allowing for subsequent data analysis.

### Descriptive statistics and correlation analysis of variables

The descriptive statistics and correlation analysis results of the variables are shown in Table 1. Parental emotional warmth is significantly negatively correlated with students’ school bullying. Parental emotional warmth is significantly positively correlated with social support. Social support is significantly negatively correlated with students’ school bullying. Social support is significantly negatively correlated with emotional regulation. The results indicate that further mediation effect analysis can be conducted.

**Table 1:** Descriptive statistics and correlation analysis results (n = 566).

Variable	M ± SD	1	2	3
1. Students’ School Bullying	1.343 ± 0.553			
2. Students’ Emotional Regulation	4.523 ± 0.837	.038		
3. Students’ Social Support	5.454 ± 1.072	-.187^**^	-.245^**^	
4. Parental Emotional Warmth	3.016 ± 0.634	-.190^**^	-.169^**^	.602^**^

Note:

* p < 0.05,

** p < 0.01,

*** p < 0.001.

### Direct effect test between parental emotional warmth and students’ school bullying

Regression analysis results showed that parental emotional warmth has a significant positive predictive effect on students’ school bullying (β = -0.138, t = -4.542, p < 0.001).

### Chain mediation effect test of social support and emotional regulation between parental emotional warmth and students’ school bullying

**Fig 1:**
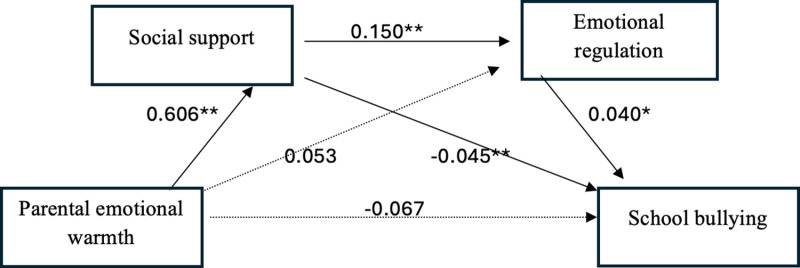
Chain mediation model.

Regression analysis results showed (as shown in Fig 1): Parental emotional warmth has a significant positive predictive effect on social support (β = 0.606, t = 17.12, p < 0.001); Parental emotional warmth has an insignificant positive predictive effect on emotional regulation (β = 0.053, t = 0.53, p = 0.603); Social support has a significant negative predictive effect on students’ school bullying (β = -0.045, t = -3.263, p = 0.001); Emotional regulation has a significant positive predictive effect on students’ school bullying (β = 0.040, t = 2.332, p = 0.024); Social support has a significant negative predictive effect on emotional regulation (β = -0.150, t = 4.322, p < 0.001); When social support and psychological health are included as mediating variables in the regression equation, the direct predictive effect of parental emotional warmth on students’ school bullying is not significant (β = -0.067, t = -1.733, p = 0.085).

The mediation effect analysis results (as shown in Table 2) indicate that the direct effect includes 0 within the 95% confidence interval, suggesting that the direct effect is not significant. The direct path effect of parental emotional warmth on students’ school bullying is 0.0665, which accounts for 50.5% of the total effect. The total mediation effect of social support and emotional regulation between parental emotional warmth and students’ school bullying does not include 0 in the 95% confidence interval, indicating that the total mediation effect is significant (accounting for 49.5% of the total effect).

**Table 2:** Mediation effect analysis of social support and emotional regulation in the relationship between parental emotional warmth and students’ school bullying.

		95% Confidence Interval	Effect Value	Effect Proportion
Direct effect	Parental Emotional Warmth → School Bullying	[-0.142, 0.009]	-0.067	50.5%
	Parental Emotional Warmth → Social Support → School Bullying	[-0.132, -0.024]	-0.078	42.6%
Mediation effect	Parental Emotional Warmth → Emotional Regulation → School Bullying	[-0.007, 0.014]	0.002	1.3%
	Parental Emotional Warmth → Social Support → Emotional Regulation → School Bullying	[0.006, 0.025]	0.010	5.7%
Total mediation effect		[-0.121, -0.011]	-0.065	49.5%
Total Effect		[-0.192, -0.071]	-0.132	100%

Parental emotional warmth mainly influences students’ school bullying through the following three mediation paths: (1) Parental emotional warmth → Social support → School bullying, with a significant mediation effect (accounting for 42.6% of the total effect); (2) Parental emotional warmth → Emotional regulation → School bullying, with a non-significant mediation effect (accounting for 1.3% of the total effect); (3) Parental emotional warmth → Social support → Emotional regulation → School bullying, with a significant mediation effect (accounting for 5.7% of the total effect). Based on the findings, the chain mediation role model of social support and emotional regulation between parental emotional warmth and students’ school bullying proposed in this study is validated.

## Discussion

This study constructed a chain mediation model involving parental emotional warmth, social support, emotional regulation, and school bullying among students to examine the internal mechanisms by which parental emotional warmth influences school bullying. The findings indicate that parental emotional warmth can affect school bullying through the mediation roles of social support and emotional regulation, as well as their chain mediation effect. Theoretically, this helps to reveal the internal mechanisms of how parental emotional warmth affects school bullying, enriching the theoretical research in this field. Practically, it provides empirical support for multi-faceted prevention and intervention efforts to reduce school bullying among students.

### The relationship between parental emotional warmth and school bullying

The results of this study show that the direct effect of parental emotional warmth on school bullying is not significant. This indicates that parental emotional warmth alone cannot directly reduce or increase bullying behavior among students. This finding aligns with some literature suggesting that the impact of family education on student behavior needs to be realized through complex psychological and social mechanisms [40]. Although emotionally warm parents can provide a safe and supportive family environment for their children, this direct influence may be insufficient to address the various complex factors in the school environment, such as peer pressure and school culture.

Parental emotional warmth education includes caring, understanding, and support for children, which help them develop a positive self-concept and self-esteem. However, these positive psychological traits may exert more influence within the family, while in the school environment, children need to cope with multiple influences from peers and the school. This requires a broader social support network and effective emotional regulation strategies to translate the positive effects of family education into reduced bullying behavior.

### The mediating role of social support in the relationship between parental emotional warmth and school bullying

The study results indicate that parental emotional warmth education has a significant mediating effect on school bullying through social support. This suggests that parental emotional warmth can indirectly reduce bullying behavior by enhancing children’s social support networks. Social support includes emotional and practical help from family, friends, and school, which can improve children’s adaptability when facing pressure and conflict [15,16]. Specifically, parental emotional warmth education can help children build positive social relationships, increasing their support networks, and enabling them to receive timely help and guidance when encountering problems.

Children with strong social support are more likely to gain emotional security and a sense of belonging, which are crucial factors in preventing bullying [41]. For example, support from friends and peers can help children find someone to confide in and seek help from when bullied, thereby reducing feelings of isolation and helplessness. Additionally, support from teachers and schools through the establishment and enforcement of anti-bullying policies can create a safer and more inclusive school environment, thereby reducing the occurrence of bullying incidents.

### The mediating role of emotional regulation in the relationship between parental emotional warmth and school bullying

Although emotional regulation is theoretically considered an important mechanism by which parenting styles influence student behavior, this study found that the mediating effect of parental emotional warmth education on school bullying through emotional regulation is not significant. This may be because the development of emotional regulation abilities is influenced by multiple factors, including personal characteristics, environmental pressures, and biological factors [22]. While parental emotional warmth can provide an environment conducive to the development of emotional regulation, this influence may take longer to manifest, and the effectiveness of emotional regulation may be limited when facing immediate conflicts and pressures in the school environment.

Furthermore, the effectiveness of emotional regulation depends on whether children can apply the learned regulation strategies in real situations. For example, even if children learn how to regulate their emotions in the family environment, the effectiveness of emotional regulation may weaken when facing actual bullying situations in school due to environmental pressures and interpersonal complexities [42]. Therefore, although emotional regulation is an important psychological ability, its mediating role between parental emotional warmth education and school bullying behavior may be limited.

### The chain mediation role of social support and emotional regulation between parental emotional warmth and school bullying

This study found that parental emotional warmth significantly influences school bullying through a chain mediation effect involving social support and emotional regulation. This indicates that parental emotional warmth can reduce bullying behavior by enhancing social support and further improving emotional regulation abilities. Specifically, social support, as an important external resource for emotional regulation, can help children better manage and regulate their emotions when facing pressure and conflict, thereby reducing aggressive behavior [43].

Research shows that children with strong social support perform better in emotional regulation because they receive more emotional and practical assistance from their support network [20]. For example, support from parents and teachers not only provides emotional comfort but also helps children adopt positive coping strategies when facing bullying, thereby reducing bullying behavior [44]. Conversely, children lacking social support may struggle with emotional regulation, increasing the risk of aggressive and bullying behavior [13].

### Implications

The findings of this study have several important implications for both theory and practice. Theoretically, the study contributes to a deeper understanding of the mechanisms through which parental emotional warmth influences school bullying. By highlighting the mediating roles of social support and emotional regulation, this research enriches the literature on the impact of family dynamics on children’s behavior and psychological well-being. Practically, the study offers actionable insights for educators, parents, and policymakers. Parents should be encouraged to foster an emotionally warm and supportive home environment, which can enhance children’s social support networks and emotional regulation abilities. Schools should implement programs that strengthen social support systems and teach effective emotional regulation strategies to students. Community-based interventions that involve parents, teachers, and peers in creating a supportive environment can also be beneficial in preventing and reducing bullying behaviors.

### Limitations and future research

Despite its contributions, this study has several limitations that should be acknowledged. First, the cross-sectional design of the study limits the ability to draw causal inferences. Future research should employ longitudinal designs to better understand the causal relationships among parental emotional warmth, social support, emotional regulation, and school bullying. Second, the reliance on self-reported data may introduce response biases, particularly social desirability bias, especially in measures like bullying and emotional warmth. To address this, future studies could incorporate multi-informant reports, objective measures, and ensure greater emphasis on confidentiality to encourage honest reporting. Third, the sample was drawn from a specific population using convenience sampling, which may limit the generalizability of the results. This sampling method restricts the external validity of the findings, and future research should include more diverse and representative populations.

## Conclusion

The findings of this study emphasize the indirect impact of parental emotional warmth education on school bullying behavior through the chain mediation roles of social support and emotional regulation. This has important implications for the development of family education and school intervention strategies. Parents should focus on providing emotional warmth and support to their children while actively helping them establish strong social support networks. Additionally, schools and communities should work together to provide comprehensive support and resources to help students develop effective emotional regulation abilities, thereby preventing and reducing school bullying behavior.
